# In this month’s Bulletin

**DOI:** 10.2471/BLT.17.000717

**Published:** 2017-07-01

Darren B Taichman et al. (482–483) present the International Committee of Medical Journal Editors’ requirements for data-sharing statements when reporting clinical trials. Timothy G Evans and Marie Paule Kieny (484) explain how health systems research is needed to inform progress towards universal health coverage.

In the news section, Apiradee Treerutkuarkul (487–488) reports on policies in Thailand to prevent alcohol-related harm. Frederico C Guanais tells Andréia Azevedo Soares (489–490) how patient empowerment can lead to improvements in health –care quality.

## Kenya

### Reducing iatrogenic infections

Guadalupe Bedoya et al. (503–517) study compliance with infection prevention and control practices.

## Liberia

### Hospitals without enough water

Nana Mensah Abrampah et al. (527–531) emphazise the need for sanitation in health care facilities. 

## Malawi

### Paying for results?

Stephan Brenner et al. (491–502) study an incentive scheme to address shortages of medical supplies and to improve use of clinical protocols.

## Nepal

### Improving use of mental health-care services

Mark JD Jordans et al. (532–536) propose ways to improve case-finding.

## Global

### Antigenic differences that matter

Hsiao-Han Chang et al. (518–526) examine genetic data for use in the design of Zika diagnostics.

### Healthy people, healthy planet

Marie Paule Kieny et al. (537–539) link sustainable development to universal health coverage. 

### No smoking or vaping

Nick Wilson et al. (540–541) list the pros and cons of banning electronic cigarettes. 

### Tracking legislation

Brooke Ronald Johnson Jr et al. (542–544) introduce a new database of national abortion laws.

